# The impact of comorbidity burden on outcomes following endovascular thrombectomy for acute ischemic stroke: A nationwide prospective observational study

**DOI:** 10.1093/esj/23969873251332136

**Published:** 2026-01-01

**Authors:** Emma Hall, Björn Hansen, Mats Pihlsgård, Magnus Esbjörnsson, Bo Norrving, Teresa Ullberg, Johan Wassélius

**Affiliations:** Medical Imaging and Physiology, Skåne University Hospital, Lund, Sweden; Department of Clinical Sciences Lund, Lund University, Lund, Sweden; Medical Imaging and Physiology, Skåne University Hospital, Lund, Sweden; Department of Clinical Sciences Lund, Lund University, Lund, Sweden; Perinatal and Cardiovascular Epidemiology, Lund University, Lund, Sweden; Department of Clinical Sciences Lund, Lund University, Lund, Sweden; Department of Neurology, Hässleholm Hospital, Hässleholm, Sweden; Department of Clinical Sciences Lund, Lund University, Lund, Sweden; Department of Neurology, Skåne University Hospital, Malmö, Sweden; Department of Clinical Sciences Lund, Lund University, Lund, Sweden; Department of Neurology, Skåne University Hospital, Malmö, Sweden; Medical Imaging and Physiology, Skåne University Hospital, Lund, Sweden; Department of Clinical Sciences Lund, Lund University, Lund, Sweden

**Keywords:** Ischemic stroke, comorbidity, endovascular thrombectomy, outcome, recanalization, complication, survival

## Abstract

**Introduction:**

Patients with substantial comorbidity burden are underrepresented in clinical trials on endovascular thrombectomy (EVT), despite being common in clinical routine care. Therefore, analysis of observational data is needed to evaluate how increasing comorbidity burden affects procedural success rate, complication rate, and clinical outcome following EVT.

**Patients and methods:**

We conducted a register-based observational study involving pre-stroke functionally independent patients treated with EVT in Sweden 2015–2021. Comorbidity burden was assessed using the Charlson Comorbidity Index (CCI) and categorized as no (CCI 0), moderate (CCI 1), severe (CCI 2), and very severe (CCI ⩾3). The primary outcome was favorable 90-day outcome (modified Rankin Scale 0–2). Secondary outcomes included successful recanalization, and peri- and postoperative complications.

**Results:**

Of 4735 included patients, 40% had no comorbidity (CCI 0), 15% had moderate (CCI 1), 21% had severe (CCI 2), and 24% had very severe comorbidity burden (CCI ⩾3). The yearly proportion of patients with very severe comorbidity burden increased from 16% to 30% during the study period. Increasing comorbidity levels were associated with decreased odds ratio (OR) of favorable outcome compared to patients without comorbidity: CCI 1 adjusted OR (aOR) 0.64, 95% CI 0.57–0.85; CCI 2 aOR 0.59, 95% CI 0.47–0.74; and CCI ⩾3 aOR 0.38, 95% CI 0.30–0.47, but there were no significant differences in successful recanalization rates. Patients with CCI 2 had higher OR for perioperative and postoperative complications (OR 1.43, 95% CI 1.09–1.88, and OR 1.41, 95% CI 1.15–1.71), and patients in the CCI ⩾3 group had higher OR of postoperative complications (OR 1.34, 95% CI 1.14–1.67), compared to patients in the CCI 0 group. Successful recanalization was associated with favorable functional outcome in all CCI-groups.

**Discussion and conclusion:**

Severe and very severe comorbidity burden are increasingly common among EVT-treated patients in routine healthcare and are linked to poorer outcomes. However, our results suggest that successful EVT is associated with functional independency, also in patients with severe and very severe comorbidity burden.

## Graphical Abstract

**Graphical abstract:**
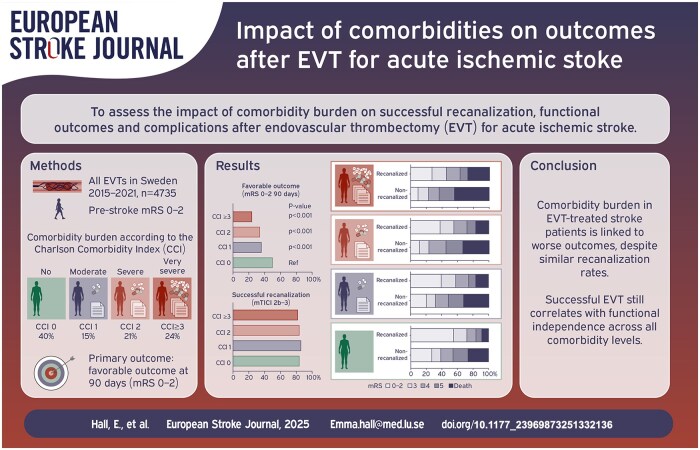

## Introduction

Endovascular thrombectomy (EVT) marked a significant shift in the management of acute ischemic stroke and has gradually become the standard of care. Recent studies have highlighted the efficacy of EVT, demonstrating substantial improvement in functional outcome and survival, even in patients with a low Alberta Stroke Program Early Computed Tomography score (ASPECTS) at presentation,^1^ as well as in patients presenting in a later time window of up to 24 h.^2,3^ However, despite the remarkable advances in treating ischemic stroke, only a small proportion of patients receive endovascular treatment.^4^

Patients with severe comorbidities have been underrepresented in randomized controlled trials assessing the efficacy of EVT in stroke. Thus, observational data are needed to evaluate how an increasing comorbidity burden impacts prognosis following EVT. Previous observational studies have shown reduced favorable outcomes following EVT in elderly patients,^5^ and in patients with cancer,^6^ metabolic syndrome,^7^ frailty,^8^ or more than one comorbidity.^9^ Multimorbidity is increasing globally as a result of prolonged life expectancy and increased survival rates from acute and chronic conditions.^10^ Several studies have linked higher comorbidity burden to increased incidence of stroke.^11,12^ However, the prevalence of comorbidities in the EVT population, and their association with patient outcome, recanalization status, and early complications is not well described in the literature. Sweden, with its public healthcare system and comprehensive national quality registers, with very high coverage of stroke, EVT, and comorbidity data, offers a unique opportunity to observationally study outcomes following EVT in stroke patients with varying comorbidities.

The aim of this study is to investigate the distribution and temporal changes of comorbidity burden in Swedish stroke patients treated with EVT over seven consecutive years. Additionally, we aim to explore associations between comorbidity burden and functional outcome, survival rates, successful recanalization, perioperative complications, and postoperative complications in acute ischemic stroke patients treated with EVT.

## Methods

### Study design

We conducted a nationwide prospective register-based observational study using data from the two Swedish quality registers for stroke care: The Swedish Stroke Register (Riksstroke)^13^ and the Swedish Endovascular Treatment of Acute Stroke (EVAS) register,^14^ as well as the Swedish Patient Register,^15^ and the Swedish Prescribed Drug Register.^16^ Data were obtained from these registers for all patients diagnosed with ischemic stroke and treated with EVT between 2015 and 2021. Exclusion criteria included spontaneous reperfusion, a history of previous EVT, unknown pre-stroke functional level, or pre-stroke functional dependency.

### Data sources

Data on pre- and post-stroke functional status and outcome were obtained from Riksstroke, which includes data from all 72 Swedish hospitals managing acute stroke care. Procedural details, stroke characteristics, treatment strategies, and complications were collected from the EVAS register, which gathers data from the six Swedish EVT centers.

Information regarding comorbidities was obtained from Riksstroke and the Swedish National Patient Register. The Swedish Patient Register is managed by the National Board of Health and Welfare and includes data on all patients receiving treatment within the Swedish healthcare system, including primary and secondary diagnoses according to the ICD-10. To increase the completeness of dementia diagnoses, filled prescriptions of medications for dementia (Anatomical Therapeutic Chemical (ATC) codes: N06DA02, N06DA03, N06DA04, and N06DX01), were also obtained from the Swedish National Prescribed Drug Register and added as a defining criterion for dementia prevalence. During the study period, the EVAS register and Riksstroke reported coverage rates of ⩾95% and ⩾85%, respectively.^13,14^ The Swedish Patient Register and the Swedish Prescribed Drug Register have a consistent ⩾98% coverage.^15,16^

### Main variables

#### Comorbidity

The Charlson Comorbidity Index (CCI) was originally developed as a prospectively applicable method to predict 1-year mortality in patients with comorbidities.^17^ The CCI provides an assessment of the total comorbidity burden by combining a selection of conditions that are weighted differently based on its individual relative risk of 1-year mortality.

In this study, we included diseases according to the original version of the CCI (Supplemental Table 1), diagnosed within 5 years prior to stroke onset. The comorbidity burden was divided into four groups: (i) no comorbidity (CCI 0); (ii) moderate comorbidity burden (CCI 1); (iii) severe comorbidity burden (CCI 2); and (iv) very severe comorbidity burden (CCI ⩾3), in line with previous studies.^12,18^

#### Radiological evaluation

Intracranial occlusions were classified as either anterior or posterior circulation occlusions. Anterior circulation occlusions were further categorized according to the proximal end of the thrombus on digital subtraction angiography (DSA) prior to EVT. I-type and T-type internal carotid artery (ICA) occlusions were both defined as ICA occlusions. Segments of the middle cerebral artery (MCA) were subdivided into M1 or M2/M3.

ASPECTS, a 10-point quantitative scoring system, assesses ischemic injury in the MCA territory, with lower scores indicating larger areas of ischemic changes.^19^ In this study, the extent of infarction was categorized into four modified ASPECTS (mASPECTS) groups based on the available infarct classification in the EVAS register: (1) mASPECTS 10: no ischemic injury; (2) mASPECT 7–9: early ischemic changes involving less than one third of the MCA territory or infarct in the basal ganglia; (3) mASPECT 5–6: infarct in less than one third of the MCA territory and infarct in the basal ganglia, and (4) mASPECTS 0–4: early ischemic changes involving more than one third of the MCA territory, or early ischemic changes in the cerebellum, pons, or medulla oblongata, since we included occlusions in the posterior circulation.

#### Successful recanalization or non-recanalization

Successful recanalization was defined as achievement of a modified Thrombolysis in Cerebral Infarction (mTICI) score of 2b–3 at the end of the EVT procedure. Non-recanalization was defined as commencement of EVT without obtaining recanalization either due to inability to access the site of occlusion, or due to a poor recanalization result defined as a mTICI score of 0–2a at the end of the procedure.

#### Functional outcome

In this study, a *favorable outcome* was defined as modified Rankin Scale (mRS), 0–2 at 90 days following stroke, which translates as the ability to carry out daily activities with no or minimal assistance. Scores of mRS 3–5 represent increasing degree of functional dependence, where mRS 5 indicates severe disability necessitating constant nursing and oversight. *Unfavorable outcome*, including death, was defined as mRS 3–6. mRS was estimated using a validated syntax based on the Riksstroke variables related to activities of daily living, self-reported by the patients, their next-of-kin, or caregiver.^20^

#### Perioperative complications

All perioperative complications registered or described as severe in EVAS were included. These complications comprised vessel perforation, hypoperfusion, perforating artery injuries, dissection, severe cardiac arrythmia, thromboembolic event in the lower extremities, defective material, severe iatrogenic arterial puncture complication, injury to a new vascular territory, and stent thrombosis.

#### Postoperative complications

Postoperative complications were categorized as follows: intracranial hemorrhage (ICH), symptomatic intracranial hemorrhage (sICH), malignant middle cerebral artery infarction, severe infections including pneumonia, and cardiovascular events. sICH was defined as an intracranial hemorrhage within 4–36 h following EVT resulting in an increase with a minimum of 4 points on the National Institutes of Health Stroke Scale (NIHSS), or death within 7 days, in line with the ECASS III definition.^21^ Cardiovascular events encompassed heart failure, cardiac arrest, pulmonary embolism, pulmonary edema, and arrhythmia.

### Primary and secondary outcomes

The primary outcome in this study was favorable functional outcome (mRS 0–2) 90 days after EVT. Secondary outcomes included 90-day survival rate, successful recanalization, as well as peri- and postoperative complications stratified by CCI group. A pre-specified sub-analysis was performed to evaluate the association between successful recanalization and a favorable 90-day outcome for each CCI group.

### Statistical analysis

Statistical analyses were conducted using IBM SPSS Statistics version 28. Baseline characteristics were presented as medians with interquartile ranges (IQR), or as proportions. A two-sided *p*-value <0.05 was considered statistically significant.

Univariable logistic regression was used to assess the relationship between comorbidity burden and four outcomes: (i) favorable functional outcome at 90 days; (ii) successful recanalization; (iii) perioperative complications; and (iv) postoperative complications. To further evaluate the association between comorbidity burden and a favorable outcome, a multivariable logistic regression analysis was conducted, adjusting for age, sex, NIHSS score before EVT, successful recanalization, time from stroke onset to groin puncture, and mASPECTS.

The association between non-recanalization and both perioperative and postoperative complications were explored using a *x*^2^ test. The association between successful recanalization and favorable 90-day functional outcome, stratified by CCI group, was examined using both univariable and multivariable logistic regression analyses, adjusting for age, sex, NIHSS score prior to EVT, time from stroke onset to groin puncture, and mASPECTS. Furthermore, a sensitivity analysis was performed excluding patients with perioperative complications from the multivariable logistic regression models assessing the relationship between successful recanalization and favorable outcome, within each CCI group.

Kaplan-Meier survival curves were used to illustrate the 90-day mortality rates, and Cox-regression with Hazard-ratio (HR) was used to analyze probability of 90-day survival. The analyses were stratified by CCI group and successful recanalization status for patients with and without perioperative or postoperative complications.

Missing mRS follow-up data were addressed using multiple imputation by chained equations with the following predicting variables: comorbidity burden, age, sex, NIHSS score before and 24 h after EVT, mTICI score at the end of the procedure, mASPECT, and use of general anesthesia during EVT. Twenty complete datasets were generated and the estimates from each imputation were combined using Rubin’s rule. Imputed data were included in both mRS illustrations and regression analyses for mRS outcome.

## Results

Of the 5291 EVT treatments performed in Sweden between 2015 and 2021, 556 patients were excluded from the study due to spontaneous reperfusion (*n* = 90), history of previous EVT (*n* = 82), unknown pre-stroke functional level (*n* = 43), or pre-stroke dependency (*n* = 341), resulting in a final study population of 4735 patients (Supplemental Figure 1).

Baseline data on patient and stroke characteristics and procedural factors are shown in Table 1. The median age was 74 years, and 46% were female. Of the 4735 included cases, 40% (*n* = 1914) had no comorbidity (CCI 0), 15% (*n* = 699) had moderate comorbidity burden (CCI 1), 21% (*n* = 975) had severe comorbidity burden (CCI 2), and 24% (*n* = 1147) had very severe comorbidity burden (CCI ⩾3). Patients with CCI 0 had the lowest median age (69 years, IQR 58–77), and patients with CCI ⩾3 had the highest (77 years, IQR 71–83; *p* < 0.001). Occlusion in the M1-segment was the most common occlusion site within all comorbidity groups (ranging from 41% to 42%). Median preoperative NIHSS score was 15 for all CCI groups, except for CCI ⩾3 where the median NIHSS score was 16. The postoperative NIHSS score 24 h after EVT was lowest for patients with CCI 1 (median 5, IQR 2–13) and highest in the CCI ⩾3 group (median 8, IQR 3–16; *p* = 0.010).

**Table 1.** Baseline characteristics of the 4735 patients included in the study stratified into groups based on comorbidity burden.

Demographics	All patients *n* = 4735	CCI 0 *n* = 1914 (40%)	CCI 1 *n* = 699 (15%)	CCI 2 *n* = 975 (21%)	CCI ⩾3 *n* = 1147 (24%)
Median age, years (IQR)	74 (65–81)	69 (58–77)	75 (68–82)	75 (68–82)	77 (71–83)
Female sex	2171 (46)	884 (46)	357 (51)	446 (46)	484 (42)
ICA occlusion	700 (15)	287 (15)	108 (15)	151 (15)	154 (13)
M1 occlusion	1962 (41)	790 (41)	286 (41)	410 (42)	476 (41)
M2/M3 occlusion	980 (21)	368 (19)	156 (22)	191 (20)	265 (23)
Posterior circulation occlusion	542 (11)	255 (13)	75 (11)	94 (10)	118 (10)
Multiple occlusions	163 (3)	70 (4)	19 (3)	39 (4)	35 (3)
Other occlusion or not specified	388 (8)	144 (8)	55 (8)	90 (9)	99 (9)
mASPECTS					
10	2228 (47)	825 (43)	371 (53)	460 (47)	572 (50)
7–9	1668 (35)	718 (38)	228 (33)	325 (33)	397 (35)
5–6	272 (6)	118 (6)	25 (4)	71 (7)	58 (5)
0–4	276 (6)	140 (7)	32 (5)	55 (6)	49 (4)
Median NIHSS score before EVT (IQR)	15 (9–19)	15 (9–19)	15 (10–19)	15 (10–20)	16 (10–20)
Median NIHSS score after EVT (IQR)	7 (2–14)	6 (1–13)	5 (2–13)	7 (2–16)	8 (3–16)
General anesthesia	1998 (42)	827 (43)	295 (42)	432 (44)	444 (39)
IVT	2205 (47)	1041 (54)	317 (45)	457 (47)	390 (34)
Recanalized (mTICI 2b–3)	3958 (84)	1599 (84)	599 (86)	819 (84)	941 (82)

CCI: Charlson Comorbidity Index; IQR: inter quartile range; ICA: internal carotid artery; M1: M1-segment of the middle cerebral artery; M2/M3: M2/M3-segment of middle cerebral artery; mASPECTS: modified Alberta Stroke Program Early CT Score; NIHSS: National Institute of Health Stroke Scale; EVT: endovascular thrombectomy; IVT: intravenous thrombolysis.

Data are presented as numbers (%) unless median (IQR) is indicated.

Missing data were ⩽1% in all variables except for mASPECT (6.7%), NIHSS score before EVT (6.9%), and NIHSS score 24 h after EVT (15.9%).

### Incidence of individual diagnoses

**Figure 1.**
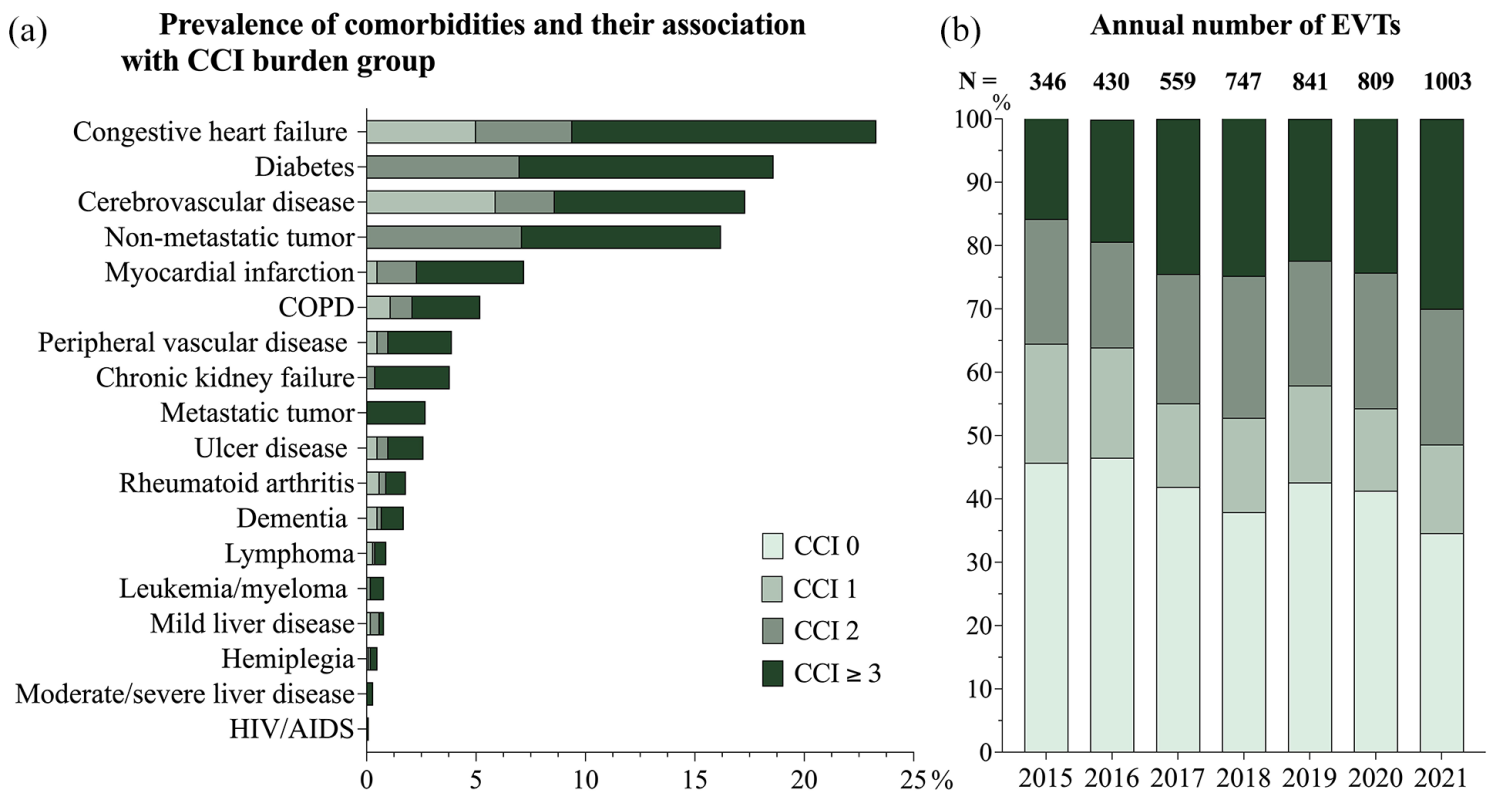
Frequency of individual diagnoses and CCI groups (a) and the annual number of performed EVTs (b). CCI: Charlson Comorbidity Index; EVT: endovascular thrombectomy; COPD: chronic obstructive pulmonary disease; AIDS: acquired immunodefiency disorder.

The frequency of comorbidities and their relation to the comorbidity burden score are illustrated in Figure 1(a). In this study, 146 patients with tumor diagnoses were recorded both with and without metastases due to the longitudinal nature of diagnosis assessments. These cases were classified as metastatic tumors. Similarly, eight patients were documented with both mild and severe liver disease, and these patients were categorized as having severe liver disease in this study.

Congestive heart failure was the most common comorbidity (23%, *n* = 1104) followed by diabetes (19%, *n* = 883). HIV/AIDS was the least common diagnosis with a total of five patients. During the observed period, there was a general increase in the yearly number of EVT treatments, from 346 in 2015 to 1,003 in 2021. As illustrated in Figure 1(b), the majority of EVT procedures were performed on patients with moderate or severe comorbidity burden. For patients with CCI ⩾3, the number of EVT treatments per year increased from 55 in 2015 to 301 in 2021, corresponding to a growth of the relative proportion from 16% to 30% (Figure 1(b)).

### Functional outcome and survival

Of the 4735 pre-stroke independent patients treated with EVT, 1809 (38%) had a favorable functional outcome (mRS 0–2) at 90 days, while 2036 (43%) were functionally dependent (mRS 3–5), and 890 (19%) had died. Hence, a total of 2835 patients (62%) experienced an unfavorable 90-day functional outcome.

**Figure 2.**
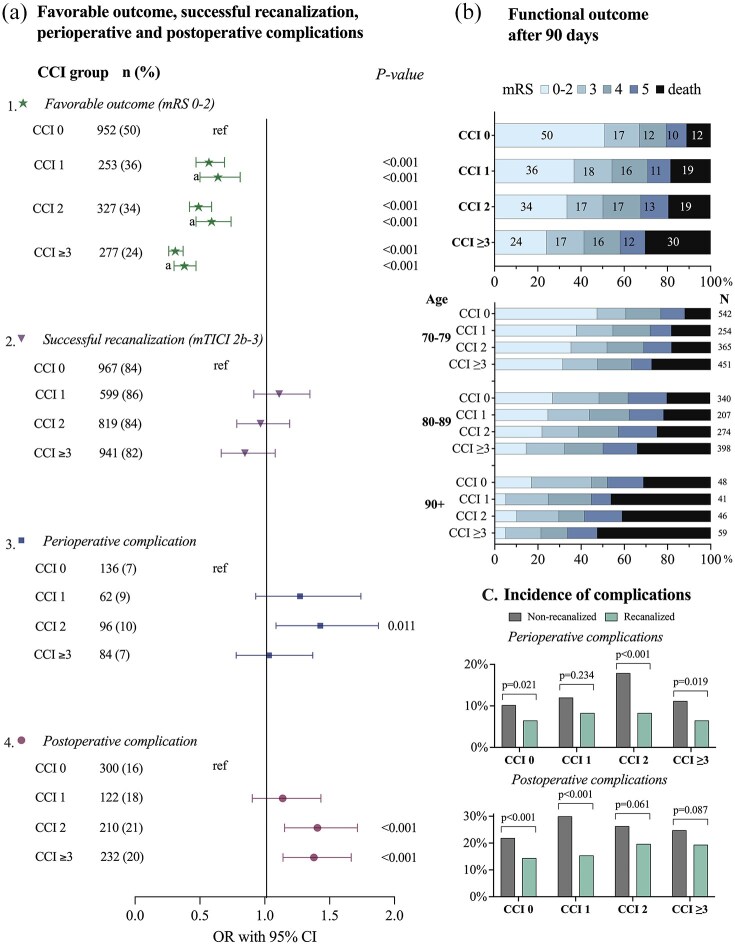
(a) (1) The association between CCI groups and favorable outcome analyzed with univariable logistic regression, and multivariable logistic regression (a) with adjustment for age, sex, NIHSS score before EVT, successful recanalization, and mASPECT. (2) The association between CCI groups and successful recanalization analyzed with univariable logistic regression. (3) The association between CCI groups and any perioperative complication analyzed with univariable logistic regression. (4) The association between CCI groups and any postoperative complication analyzed with univariable logistic regression. (b) mRS outcome after 90 days. (c) The incidence of peri- or postoperative complications for recanalized and non-recanalized patients stratified by CCI groups. CCI: Charlson Comorbidity Index; mRS: modified Rankin Scale; mTICI: modified thrombolysis in cerebral infarction; OR: odds ratio; CI: confidence interval.

There was a strong association between increasing comorbidity burden and decreasing proportions achieving a favorable 90-day functional outcome post EVT in the univariable regression analysis. Here, 50% of patients in the no comorbidity group had a favorable outcome at 90 days, compared to 36% in the CCI 1 group (OR: 0.57, 95% CI 0.47–0.69), 34% in the CCI 2 group (OR: 0.49, 95% CI 0.42–0.59), and 24% in the CCI ⩾ 3 group (OR: 0.31, 95% CI 0.26–0.37; Figure 2A(a) and (b)). The significant association between increasing comorbidity burden and decreasing rate of favorable outcome 90-days after EVT remained across all comorbidity groups compared to the group without comorbidities in the multivariable regression analyses (CCI 1: adjusted OR (aOR) 0.64, 95% CI 0.50–0.81; CCI 2: aOR 0.59, 95% CI 0.47–0.74; and CCI ⩾ 3: aOR 0.38, 95% CI 0.30–0.47), adjusting for age, sex, NIHSS score, successful recanalization, time from stroke onset to groin puncture, and mASPECTS.

The distribution of 90-day mRS outcomes (Figure 2(b)) revealed a progressive decline in favorable outcome with each increment in CCI burden group. At 90 days post EVT, a total of 3845 patients (81%) were alive. Stratified by comorbidity group, the survival rate was 88% (*n* = 1691) for patients with no comorbidities (CCI 0), 81% (*n* = 569) for patients in the moderate comorbidity group (CCI 1), 81% (*n* = 786) in the severe comorbidity group (CCI 2), and 70% (*n* = 799) in the very severe comorbidity group (CCI ⩾3). The differences between the comorbidity groups remained but decreased after stratifying patients by age groups (70–79 years, 80–89 years, and >89 years). However, in the oldest age group, the CCI 1 group showed lower rates of mRS 0–2 and higher rates of mRS 6 compared to the CCI 2 group.

### Successful recanalization

From the whole study population, 84% (*n* = 3958) of patients were successfully recanalized (mTICI 2b–3) and 16% (*n* = 777) were not (mTICI 0–2a). The rate of successful recanalization was highest in the group with moderate comorbidity burden (CCI 1: 86%) and lowest in the very severe comorbidity burden group (CCI ⩾3: 82%). There was no difference in odds for successful recanalization between the comorbidity groups analyzed with univariable logistic regression with CCI 0 as reference (CCI 1: OR 1.18, 95% CI 0.93–1.51; CCI 2: OR 1.03, 95% CI 0.8–1.23; CCI ⩾3: OR 0.9, 95% CI 0.74–1.09, Figure 2(a)). Baseline demographics, stroke characteristics, and procedural details for successfully recanalized and non-recanalized patients are presented in Table 2. There were no, or small, differences in median age between recanalized and non-recanalized patients across all CCI groups. The proportion of females was slightly higher for non-recanalized patients within the CCI 1 group. The median NIHSS score after EVT was lower or equal to the median NIHSS score before EVT within all comorbidity groups, for recanalized as well as non-recanalized patients.

**Table 2.** Baseline characteristics of successfully recanalized and non-recanalized patients included in the study stratified into groups based on comorbidity burden.

CCI burden group	Successfully recanalized	Non-recanalized	*p*-Value
*No comorbidity, CCI 0*	1599 (84)	315 (17)	
Median age, years (IQR)	68 (58–77)	71 (61–80)	0.006
Female sex	740 (46)	144 (46)	
ICA occlusion	245 (15)	42 (13)	
M1 occlusion	678 (42)	112 (36)	0.024
M2/M3 occlusion	296 (19)	72 (23)	
Posterior circulation occlusion	212 (13)	43 (14)	
mASPECTS			0.074
10	704 (49)	121 (42)	
7–9	592 (41)	126 (44)	
5–6	98 (3)	19 (7)	
0–4	108 (7)	32 (11)	
Median NIHSS score before EVT (IQR)	15 (9–19)	14 (9–19)	
Median NIHSS score after EVT (IQR)	5 (1–12)	13 (6–18)	<0.001
General anesthesia	719 (45)	108 (34)	<0.001
IVT	880 (55)	161 (51)	
*Moderate comorbidity burden, CCI 1*	599 (86)	100 (14)	
Median age, years (IQR)	75 (68–82)	77 (68–83)	
Female sex	302 (50)	55 (55)	
ICA occlusion	93 (16)	15 (15)	
M1 occlusion	256 (43)	30 (30)	0.016
M2/M3 occlusion	125 (21)	31 (31)	0.024
Posterior circulation occlusion	62 (10)	13 (13)	
mASPECTS			0.790
10	320 (58)	51 (38)	
7–9	194 (35)	34 (38)	
5–6	23 (4)	2 (2)	
0–4	27 (5)	5 (5)	
Median NIHSS score before EVT (IQR)	15 (10–19)	14 (8–19)	
Median NIHSS score after EVT (IQR)	5 (1–12)	11 (7–19)	<0.001
General anesthesia	262 (44)	33 (33)	0.044
IVT	273 (46)	44 (44)	
*Severe comorbidity burden, CCI 2*	819 (84)	156 (16)	
Median age, years (IQR)	75 (68–82)	76 (68–82)	
Female sex	377 (46)	69 (44)	
ICA occlusion	126 (15)	25 (16)	
M1 occlusion	363 (44)	47 (30)	<0.001
M2/M3 occlusion	156 (19)	35 (22)	
Posterior circulation occlusion	77 (9)	17 (11)	
mASPECTS			0.041
10	394 (52)	66 (49)	
7–9	278 (37)	47 (35)	
5–6	56 (7)	15 (11)	
0–4	40 (5)	15 (12)	0.014
Median NIHSS score before EVT (IQR)	16 (11–20)	15 (9–19)	
Median NIHSS score after EVT (IQR)	6 (2–15)	15 (8–19)	<0.001
General anesthesia	360 (44)	72 (46)	0.613
IVT	392 (48)	65 (42)	
*Very severe comorbidity burden, CCI* ⩾*3*	941 (82)	206 (18)	
Median age, years (IQR)	77 (71–83)	78 (69–84)	
Female sex	392 (42)	92 (45)	
ICA occlusion	129 (14)	25 (12)	
M1 occlusion	410 (44)	66 (32)	0.002
M2/M3 occlusion	208 (22)	57 (28)	
Posterior circulation occlusion	95 (10)	23 (11)	
mASPECTS			0.949
10	469 (53)	101 (52)	
7–9	322 (37)	75 (39)	
5–6	48 (6)	10 (5)	
0–4	41 (5)	8 (4)	
Median NIHSS score before EVT (IQR)	16 (11–20)	15 (9–19)	
Median NIHSS score after EVT (IQR)	7 (2–14)	15 (8–19)	<0.001
General anesthesia	384 (41)	60 (29)	0.002
IVT	314 (33)	76 (37)	

CCI: Charlson Comorbidity Index; IQR: inter quartile range; ICA: internal carotid artery; M1: M1-segment of the middle cerebral artery; M2/M3: M2/M3-segment of middle cerebral artery; mASPECTS: modified Alberta Stroke Program Early CT Score; NIHSS: National Institute of Health Stroke Score; EVT: endovascular thrombectomy; IVT: intravenous thrombolysis.

Data are presented as numbers (%) unless median (IQR) is indicated.

Missing data were ⩽1% in all variables except for mASPECT (6.7%), NIHSS score before EVT (6.9%), and NIHSS score 24 h after EVT (15.9%).

### Perioperative and postoperative complications

The overall incidence of any perioperative complication was 8% (*n* = 378) in the whole study population, and the total incidence of postoperative complications was 19% (*n* = 885). Patients in the CCI 0 group had the lowest incidence of both perioperative and postoperative complications (7% and 16%, respectively). These compare with 9% and 18% in the CCI 1 group, 10% and 21% in the CCI 2 group, and 7% and 20% in the CCI ⩾3 group. The CCI 2 group had higher odds of perioperative complications in the univariable logistic regression analyses, with CCI 0 as reference (Figure 2(a)). In the univariable logistic analyses for postoperative complications there was a significant association for CCI 2 group and CCI ⩾3 group, when compared to the CCI 0 group (Figure 2(a)).

The incidence of perioperative and postoperative complications was lower in successfully recanalized than in non-recanalized patients across all CCI groups (Figure 2(c)). Among patients with successful recanalization, the incidence of perioperative complications ranged from 7% (CCI 0, CCI ⩾3) to 8% (CCI 1, CCI 2), while postoperative complications occurred in 14% (CCI 0)–20% (CCI 2) of patients. In non-recanalized patients, the incidence of perioperative complications ranged from 10% (CCI 0) to 18% (CCI 2), and postoperative complications occurred in 22% (CCI 0) to 30% (CCI 1) of patients. The lowest frequency of both perioperative and postoperative complications was observed in the CCI 0 group, regardless of recanalization status.

The prevalences of individual perioperative or postoperative complications within the CCI groups are further described in Supplemental Table 2.

### Successful recanalization and functional outcome

**Figure 3.**
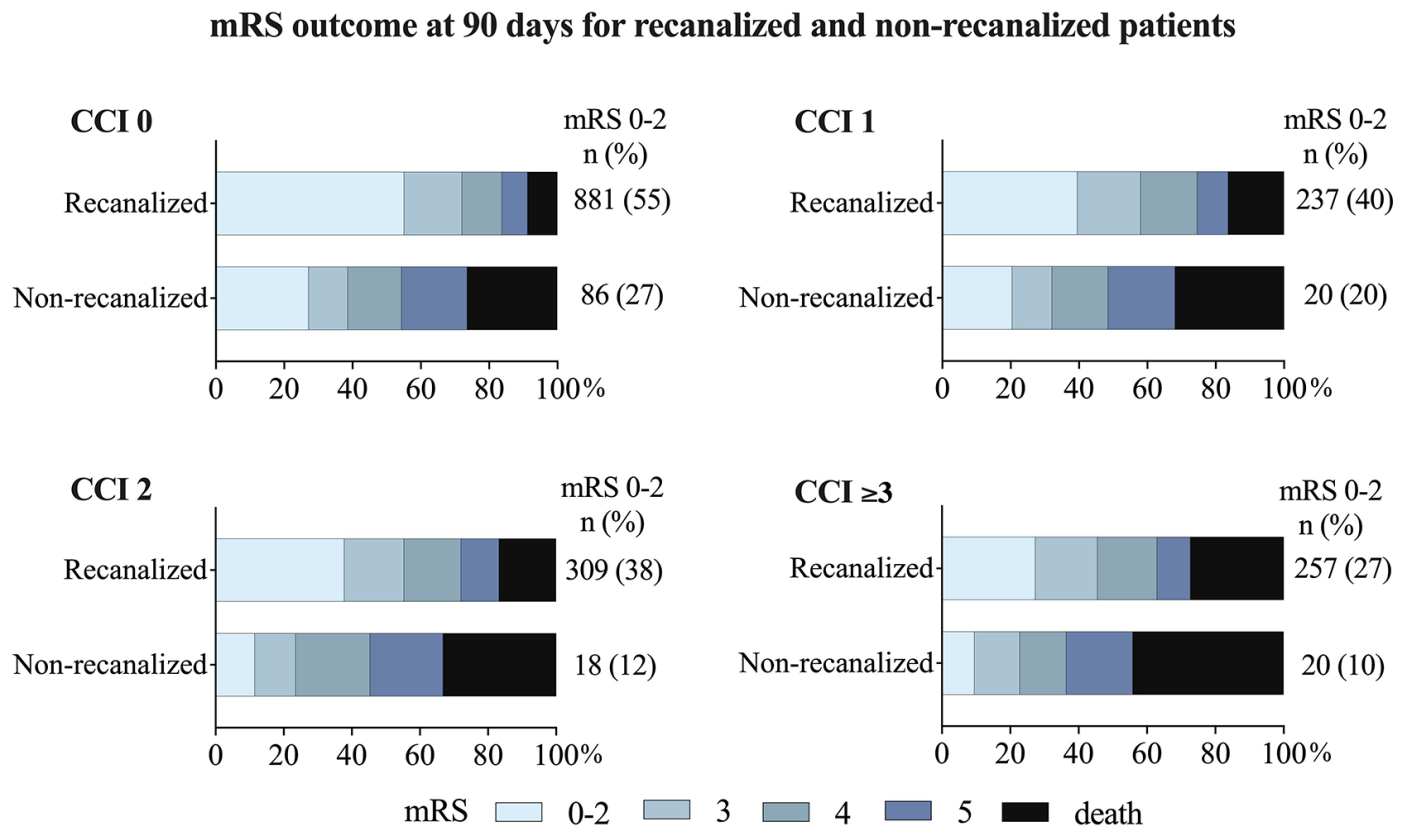
mRS outcome in recanalized and non-recanalized patients, in relation to pre-existing comorbidity burden groups. CCI: Charlson Comorbidity Index; OR: odds ratio; mRS: modified Rankin Scale; mRS 0–2: favorable functional outcome.

Successful recanalization was associated with increased survival rates and functional independence within all comorbidity groups (Figure 3). The proportion of favorable outcome in patients with successful recanalization ranged from 55% (CCI 0) to 27% (CCI ⩾ 3). In patients without successful recanalization, the proportion of favorable outcomes ranged from 27% (CCI 0) to 10% (CCI ⩾3). The OR for favorable outcome decreased with increasing comorbidity burden both for recanalized and for non-recanalized patients. There was a significant association between successful recanalization and a favorable outcome in all comorbidity groups even when adjusting for age, sex, NIHSS score before EVT, time from stroke onset to groin puncture, and mASPECTS (CCI 0: aOR 3.42,95% CI 2.41–4.83, *p* < 0.001; CCI 1: aOR 3.34, 95% CI 1.71–6.49, *p* < 0.001; CCI 2: aOR 6.00, 95% CI 3.00–10.83, *p* < 0.001; and CCI ⩾3: aOR 4.21, 95% CI 2.92–7.41, *p* < 0.001). Severe disability (mRS 5) was more than twice as common in non-recanalized compared to recanalized patients, across all comorbidity groups. This includes the group with very severe comorbidity burden (CCI ⩾3) where the proportion of mRS 5 was 19% in non-recanalized patients and 9% in recanalized patients.

#### Sensitivity analysis

After excluding the 378 patients with perioperative complications from the multivariable logistic regression analysis, the significant associations between successful recanalization and favorable outcomes remained across all CCI groups, CCI 0: aOR 3.45 (95% CI 3.24–3.68, *p* < 0.001); CCI 1: aOR 3.03 (95% CI 2.67–3.44, *p* < 0.001); CCI 2: aOR 5.46 (95% CI 4.85–6.15, *p* < 0.001); and CCI ⩾3: aOR 4.12 (95% CI 3.68–4.60, *p* < 0.001).

### Survival in relation to recanalization status and peri- and postoperative complications

**Figure 4.**
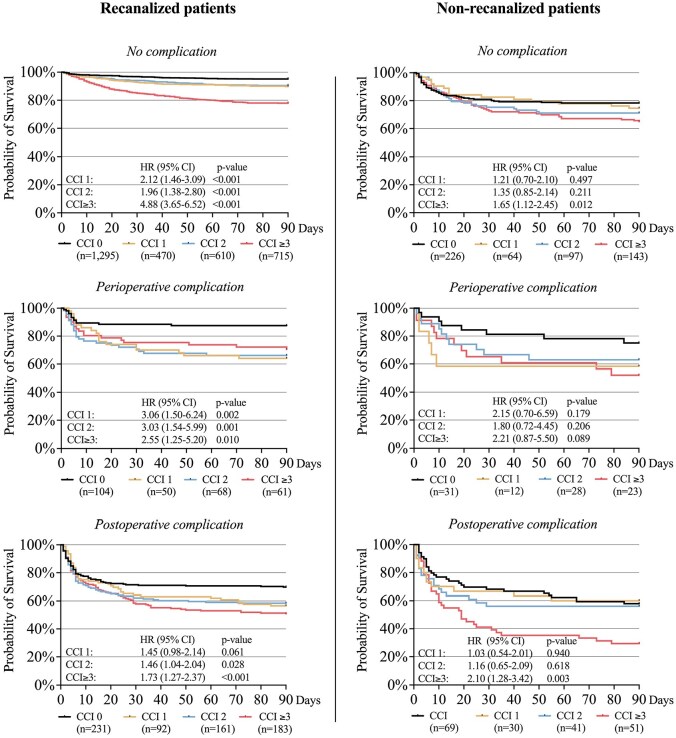
Kaplan-Meier curves illustrating the survival rate for patients with and without successful recanalization, perioperative complications, and postoperative complications, stratified by comorbidity burden groups. CCI: Charlson Comorbidity Index; HR: hazard-ratio.

Mortality data were complete for all 4735 patients, allowing their inclusion in the Kaplan-Meier curves for 90-day survival (Figure 4). These curves illustrate the difference in survival rates based on the presence or absence of successful recanalization, perioperative complications and postoperative complications across the comorbidity burden groups, including HR for probability of 90-day survival. The curves revealed decreased survival rate for patients with perioperative complications, regardless of recanalization status, across CCI groups, and even lower survival rates for patients with postoperative complications. The survival rates were higher for recanalized compared to non-recanalized patients across all CCI groups, irrespective of complication status.

## Discussion

In this nationwide study spanning seven consecutive years, the proportion of EVT-treated patients with very severe comorbidity burden (CCI ⩾3) nearly doubled, increasing from 16% to 30% of the total EVT population. While patients with higher comorbidity burden had a worse prognosis compared to patients with no or low comorbidity burden – particularly in cases without successful recanalization and/or perioperative or postoperative complications – our results suggest that pre-stroke functionally independent patients at all levels of comorbidity benefit from successful EVT.

Our study only includes procedures performed from 2025 to 2021. After 2021, the number of EVTs in Sweden has continued to increase in all regions, reaching 1,500 EVTs during 2023, corresponding to 10% of all ichemic strokes.^22^ This is likely due to a larger and more diverse proportion of the stroke population now receiving EVT, as highlighted in the annual report.^23^

Despite similar rates of successful recanalization across comorbidity groups (with highest percentage observed in the CCI 1 group), we observed a decrease in favorable outcome with increasing comorbidity burden, in line with previous findings.^24^ The decrease in favorable outcome was primarily attributed to an increased mortality rate within higher comorbidity groups, while the differences in mRS 3, 4, and 5 were smaller (Figure 2(b)).

An encouraging finding in our study is the observed association between successful recanalization and improved survival and functional independency for all comorbidity burden groups compared to non-recanalized counterparts, even when adjusted for age, sex, NIHSS score, and early ischemic changes on computed tomography (CT) scans. It is important to acknowledge both that this applies to pre-stroke functionally independent patients, as well as the possibility that non-recanalized patients may have experienced adverse effects from the unsuccessful EVT procedure. However, previous studies showed no difference in outcome between failed recanalization and best medical treatment,^25^ even for patients with low ASPECTS.^26^ Furthermore, our results indicated no neurological deterioration, as measured by the difference between pre- and post-EVT NIHSS score, in non-recanalized patients in any comorbidity group. This implies that the observed difference in favorable functional outcome between recanalized and non-recanalized patients may be interpreted as an estimation of treatment effect. It is, however, important to acknowledge that this is an observational study, and randomized controlled trials are necessary to asses the efficacy of EVT for patients with multiple comorbidities. The difference in prognosis between successfully recanalized and non-recanalized patients is mainly attributed to a decrease in mortality and a decrease in severe functional disability (mRS 5) in recanalized subjects. Although the threshold of an acceptable quality of life may be subjective, severe functional disability often results in social and emotional isolation, loss of autonomy, and significantly reduced quality of life. A reduction of severe functional disability (mRS 5) might therefore be regarded as at least as important as a reduction in mortality.^27^ Our results indicate that successful recanalization reduces the proportion of patients with a mRS score of 5 following EVT, across all comorbidity groups. In addition to the direct benefits for individual patients, this may have broader cost-effectiveness implications for the health system and society. This was however not analyzed in our study.

In our study, perioperative and early postoperative complications were associated with a reduced survival rate in both recanalized and non-recanalized patients. The negative impact on survival was most pronounced for patients with postoperative complications. Notably, these complications were often unrelated to the EVT procedure itself, instead representing common medical complications in hospitalized patients, such as severe infections. This suggests that severe postoperative events in multicomorbid patients occur regardless of endovascular treatment, and the findings emphasize that implementing measures to reduce the risk of postoperative complications needs to be a key priority for all EVT patients. Survival rates were lower for recanalized patients with perioperative complications compared to non-recanalized patients without complications, across all CCI groups, suggesting that the risk of perioperative complications should not be a reason to withhold treatment, even for patients with severe comorbidity burden.

### Limitations

Our study has several limitations. First, data were obtained from prospectively collected national quality registers. In EVAS, CT findings, mTICI, and the presence of any peri- or postoperative complication are reported either by the performing operator or a center without core-lab adjudication. The lack of central adjudication of radiological outcomes may have led to an overestimation of recanalization rates and underestimation of complication rates.^28^

Second, the non-recanalized group consists of patients with failed EVT. Patients eligible, but not accepted, for EVT are not identified in the Swedish quality registers, which may have caused selection bias. Thus, the results may not reflect real world multicomorbid patients for whom the risk of perioperative and postoperative complications could be even higher, and functional independency at 90 days even lower.

Third, functional outcome is self-reported by patients in Riksstroke. However, a previous validation study found good agreement between mRS estimated in Riksstroke and nurse-adjudicated mRS.^20^ Previous analyses on loss to follow-up have shown that older patients with higher dependency levels are less likely to return the follow-up questionnaire,^29^ and in our study, a total of 21% of patients were alive at 90 days but lost to follow-up. Multiple imputations with several predictive values were used to handle missing data. Still, attrition bias may result in a slight overestimation of favorable outcomes.

Fourth, some diagnoses, especially dementia, are underdiagnosed in the National Patient Register since it mostly covers hospital-based inpatient and specialized outpatient care, and many diagnoses (including dementia) are handled only in the primary care sector. The reported frequencies of comorbidities are likely lower than the true frequencies. For dementia, this was handled by obtaining the diagnosis from multiple sources (outpatient register, inpatient register, and filled prescription for dementia-specific drugs).

Fifth, information on patients’ do-not-resuscitate, do-not-intubate, and allow natural death preferences was not available in the registries. If these patients were more common in the high-comorbidity groups and received treatment limitations, this may have led to higher mortality and a weaker treatment effect in these groups.

Last, the CCI does not account for several comorbidities commonly associated with stroke, such as hypertension and atrial fibrillation, potentially leading to an underestimation of the overall comorbidity burden in this study. However, to the best of our knowledge, no well-validated comorbidity scale for stroke currently includes these conditions. Given the previously reported association between hypertension and poor functional outcomes and increased mortality,^30^ the development of a more stroke-adapted comorbidity scale that includes hypertension should be considered in future studies.

## Conclusion

We found that EVT for acute ischemic stroke in patients with a high comorbidity burden has become increasingly common in routine clinical practice. High comorbidity burden in these patients was frequently associated with higher incidence of perioperative and postoperative complications, and reduced functional independence following EVT, despite similar rates of successful recanalization when compared to patients with no or low comorbidity burden.

However, regardless of comorbidity burden, successful recanalization was associated with significantly improved odds of achieving a favorable 90-day functional outcome in pre-stroke functionally independent patients, in particular by a substantial reduction of the proportion of patients with severe disability (mRS 5). Our results highlight the need to minimize peri- and postoperative complications, especially in patients with substantial comorbidity burden, and encourage future pragmatic randomized controlled trials and cost-effectiveness research focusing on patients with multiple or severe comorbidities.

## Supplementary Material

sj-docx-1-eso_23969873251332136

## Data Availability

Anonymized data not published within this article will be made available from qualifies investigator upon reasonable request. This study follows the STROBE reporting guideline (Strengthening the Reporting of Observational Studies in Epidemiology).

## Correction (August 2025):

Article updated; for further details please see the Article Note at the end of the article.

## Article Note

The following updates were made to this article: On page 9, column 2 onwards, Citations 22-27 were incorrect and have been corrected to 24-29 to match the correct references.
